# Impact of exercise on energy metabolism in anorexia nervosa

**DOI:** 10.1186/2050-2974-1-37

**Published:** 2013-09-04

**Authors:** Stephan Zipfel, Isabelle Mack, Louise A Baur, Johannes Hebebrand, Stephen Touyz, Wolfgang Herzog, Suzanne Abraham, Peter SW Davies, Janice Russell

**Affiliations:** 1 Department of Psychosomatic Medicine & Psychotherapy, University Medical Hospital Tuebingen, Osianderstr. 5, 72074 Tuebingen, Germany; 2 Department of Psychological Medicine, University of Sydney, Sydney, Australia; 3 Department of Paediatrics & Child Health, University of Sydney, Sydney, Australia; 4 School of Psychology and Discipline of Psychiatry, University of Sydney, Sydney, Australia; 5 Department of Obstetrics & Gynaecology, University of Sydney, Sydney, Australia; 6 Eating Disorders Unit, Northside Clinic, Greenwich NSW 2065, Australia; 7 Department of General Internal and Psychosomatic Medicine, University of Heidelberg, Heidelberg, Germany; 8 Department of Child & Adolescent Psychiatry, Univ. of Essen, Essen, Germany; 9 Children’s Nutrition Research Centre, School of Medicine, University of Queensland, Brisbane, Australia

**Keywords:** Anorexia nervosa, Exercise, Energy expenditure, Physical activity, Doubly labelled water, Calorimetry

## Abstract

**Background:**

Excessive physical activity is one of the most paradoxical features of anorexia nervosa (AN). However, there is individual variation in the degree of physical activity found in AN-patients. As a result, marked differences in energy expenditure may be expected. Furthermore, exercise has a positive impact on a variety of psychological disorders and the psychopathology may be different in AN displaying high exercise levels versus AN displaying low exercise levels. We analyzed the energy metabolism and psychological data in low-level exercise and high-level exercise AN-patients compared with healthy, age matched controls.

Physical activity, energy expenditure (EE) by the doubly labelled water technique and indirect calorimetry, hormone status as well as psychopathology by questionnaires for eating disorders (EDI-SC, EDI-2), eating attitude (EAT) and depression (BDI) were assessed in twelve AN patients and twelve controls.

**Results:**

REE was decreased in AN-patients compared with controls but not when adjusted for body surface area or lean body mass. No differences in TDEE between AN- patients and controls were observed. Subgroup analyses showed that the percentage of high-level AN- exercisers was higher compared with controls. This subgroup had increased resting EE, total daily EE and scored higher on depression and the EDI-item “Drive for thinness” compared with low-level AN-exercisers.

**Conclusions:**

We identified a significant subgroup of high-level AN-exercisers (66%) with consecutive increased energy requirements. An easy way for clinicians to assess the amount of exercise before and in the course of treatment is a single question in the established Eating Disorder Inventory-SC (EDI-SC).

## Background

Anorexia nervosa (AN) is a serious illness associated with a chronic course and high mortality [1]. Excessive physical activity has been mentioned by both Lasègue, 1873 [2] and Gull, 1874 [3] as one of the most paradoxical features of AN. The prevalence of hyperactivity in AN lies between 31 and 80%, depending on the type of eating disorder and the study and its criteria for hyperactivity [4,5]. Physical activity is a metabolically expensive process. Thus, hyperactivity might be one of the underlying problems in the process of weight restoration and maintenance. However, for clinicians it is difficult to assess physical activity due to rather accurate but time-expensive methodologies or underreporting in self-assessment instruments [5,6]. Thus, often only the dietary intake is assessed resulting in false estimation of the patient’s energy requirements [7]. A simple tool for clinicians to estimate the degree of physical activity would be helpful to solve this problem.

The gold standard for assessing physical activity involves the use of the doubly labelled water technique [8] which measures the total daily energy expenditure (TDEE). TDEE consists of three components: resting energy expenditure (REE), diet induced thermogenesis (DIT) and the energy cost of physical activity [9]. REE is generally reduced in emaciation due to the decreased body weight, lean body mass (LBM) and metabolic adaptations [10-12]. In contrast, TDEE, measured by doubly labelled water, is sometimes reported to be decreased in AN and sometimes to be similar to healthy controls [12]. Additionally, we have shown previously that energy metabolism is altered in the different stages of illness; for example, early refeeding of AN is accompanied with a paradoxically high DIT [13,14]. Fat oxidation during moderate exercise is not suppressed in AN patients where BMI < 16.5 as it is in healthy controls [15]. Interestingly, the former studies on energy expenditure in AN did not distinguish between patients with high-activity levels and patients with low-activity levels and did not combine these measurements with assessing psychological data. Given the interindividual differences in activity of AN patients, we compared the energy metabolism, endocrine parameters as well as psychological data of AN patients with healthy controls and performed subgroup analysis by distinguishing between low-level and high-level exercisers.

## Methods

### Study population

Twelve consecutive female AN patients meeting criteria for AN according to the DSM-IV (APA 1994) which also includes the existence of amenorrhea. with n = 8 (67%) from the restricting subtype and n = 4 (33%) from the binge eating/purging type, were studied at the University of Sydney/Australia. Patients with comorbid physical illness or an additional axis-II disorder were excluded, as were those taking medications other than calcium supplements (n=3) and oral contraceptives (n=5). AN patients with comorbid depression were not excluded. Participants were instructed to continue their usual activities during the period of participation in this study.

All participants were in a nutritional rehabilitation program. Patients were asked to take part in the study not before an initial two weeks of stabilization. Initially they were encouraged to eat three meals and three snacks per day selected from a cafeteria situation and containing 4190–6280 kJ initially, increasing towards 10470–16750 kJ per day in the latter part of treatment. In the usual inpatient diet, 50% of the energy is derived from carbohydrate, 30% from fat and 20% from protein. Patients were fully ambulant but were required to rest on their beds for 30 minutes after breakfast and dinner and 60 minutes after lunch.

Controls (N = 12) were healthy, post pubertal, non pregnant females and in the follicular phase of the menstrual cycle. Eating disorders, dietary restriction and over-exercise were excluded by means of a careful standardized DDE interview [16]. All participants gave informed consent to the procedure and parental consent was also obtained for subjects less than 18 years of age. The protocol for the study was approved by the Human Ethics Committees of both Ramsay Healthcare and the University of Sydney, Australia.

### Protocol

After an overnight fast, all participants (patients and controls) were first interviewed, using a structured interview [16] for the assessment of an eating disorder, including a detailed history of the exercise behaviour. They then completed structured self administered questionnaires for depression (Beck depression inventory, BDI [17]), eating disorder (Eating Disorders Inventory-Symptom Checklist, EDI-SC; Eating Disorders Inventory-II, EDI-II [18]) and eating attitude (Eating Attitudes Test, EAT-40 [19]). A fasting blood sample for the analysis of leptin, thyroid hormones and basal cortisol was collected. Body composition, REE and TDEE were assessed.

### Blood samples

Fasting blood samples were immediately centrifuged and stored at −80°C. The radioimmunoassay (RIA) method was used to measure leptin (Mediagnost, Tuebingen, Germany; intra-assay variance: 5%; interassay variance: 7.6%) and cortisol (100T kit of the Nichols Institute Diagnostics, San Juan Capistrano, CA, USA; sensitivity: 0.8 μg/l, intra-assay variance: 4%, inter-assay variance: 8%). Triiodothyronine (T_3_), thyroxine (T_4_) and thyroid-stimulating hormone (TSH) were measured by chemiluminiscence (ACS Centaur, Chiron Diagnostics, Fernwald, Germany; intra-assay variance: T_3_ 2.8%; T_4_ 3.1%; TSH 2.3%; inter-assay variance: T_3_ 3.7%; T_4_ 2.7%; TSH 3.6%).

### Anthropometry

Body mass index (BMI) was calculated as weight/height^2^ (kg/m^2^). Body composition was assessed via skinfold thickness by a caliper at four sites of the body. Subsequently, the body fat mass was derived from the equations of Durnin and Womersley [20]. LBM was calculated as the difference between body mass and body fat mass. Bioelectrical impedance analysis (BIA) was measured using the body composition analyzer BIA-101 (RJL system, Detroit) in order to estimate total body water.

### Resting energy expenditure (REE)

A Datex Deltatrac II Metabolic Monitor (Datex, Finland) was used to measure REE over a 30 minute episode by indirect calorimetry. Participants were asked to lie still and were permitted to read or listen to music via headphones. They were prevented from sleeping.

### Total daily energy expenditure (TDEE)

TDEE was measured over 15 days by the doubly labelled water method and was applied as described in detail elsewhere [8,21,22]. The amounts of labelled water taken were scaled according to estimated total body water (TBW): H_2_^18^O, 0.25 g/kg TBW; ^2^H_2_0, 0.1 g/kg TBW (for details see [23]) This method, which has been shown to be accurate within a range of 5 percent in adults under free living conditions, is based on the prediction of carbon dioxide production from the differential disappearance rates of two stable isotopes [8]. Energy as activity was calculated by the equation: The Physical activity level (PAL) was assessed by the equation: PAL = TDEE/REE.

### Data analysis

All results are presented as mean and standard deviations (SD). The data were evaluated using SPSS for Windows. To compare group differences, student’s t-tests were performed. In case of skew distribution or inhomogeneous variance, Wilcoxon 2-sample-tests were computed. Rank correlations (Spearman correlation coefficient) were used to determine the degree of relationships between TDEE and BDI scores and the items “Drive for thinness” from the EDI-2. Data were adjusted for multiple testing using the Bonferroni correction.

## Results

Baseline demographic and physical characteristics of AN patients and controls are presented in Table 1. The BMI and body composition (fat mass and LBM) were significantly reduced in AN patients compared with age-matched female controls. The daily amount of exercise did not statistically differ between the groups due to the great interindividual differences as assessed by structured interview (DDE). Consequently, we differentiated between high-level exercisers and low-level exercisers by using a cutoff set at < 50% according to the EDI-SC item “What percentage of your exercise is aimed at controlling your weight?” There was a significantly higher percentage of high-level exercisers in the AN participants compared with the controls.

**Table 1:** Baseline demographic and physical characteristics of AN patients and controls

**Variable**	**Anorexia nervosa (AN)**	**Controls**		***P***
	**n = 12**	**n = 12**		
	**M ± SD**	**M ± SD**		
Age *(yrs.)*	21.9 ± 6.2	25.3 ± 3.5		n.s.
Duration of illness (yrs.)	4.3 ± 3.9			
Body mass index *(kg/m*^*2*^*)*	15.4 ± 1.0	21.8 ± 2.5		<0.001
Fat mass *(kg)*	6.4 ± 2.5	17.9 ± 4.5		<0.001
Lean body mass *(kg)*	34.3 ± 4.1	43.5 ± 4.5		<0.001
Exercise (min/day)	83.2 ± 63.9	44.2 ± 25.5		*0.06*
Exercise intensity*				0.031
High intensity, n (%)	7 (58.3%)	2 (16.7%)		
Low intensity , n (%)	5 (41.7%)	10 (83.3%)		
	**AN High exercisers**	**AN Low exercisers**	**Controls**	
	**n = 7**	**n = 5**	**n = 12**	
	**M ± SD**	**M ± SD**	**M ± SD**	
BMI (kg/m^2^)	15.9 ± 1.2	15.0 ± 0.85	21.8 ± 2.5	1c, 2c
Lean body mass *(kg)*	35.3 ± 5.1	33.5 ± 3.2	43.5 ± 4.5	1c, 2c
Exercise (min/day)	128.6 ± 51.7	33.0 ± 23.8	44.2 ± 25.5	1c, 3b

Values are expressed as means (M) ± standard deviations (SD). *From EDI-SC (“What percentage of your exercise is aimed at controlling your weight >50% cutoff”). a = *P*<0.05, b = *P*<0.01, c = *P*<0.001, n.s. = not significant.

1 = AN high exercisers vs. controls, 2 = AN low exercisers vs. controls, 3 = AN low exercisers vs. AN high exercisers.

The amount of daily exercise in patients labeled as high-level exercisers was 3- to 4-fold higher compared with the controls and low-level exercisers, respectively. Despite the varying activity levels in the AN subgroups, no differences in BMI or LBM were observed.

**Figure 1:**
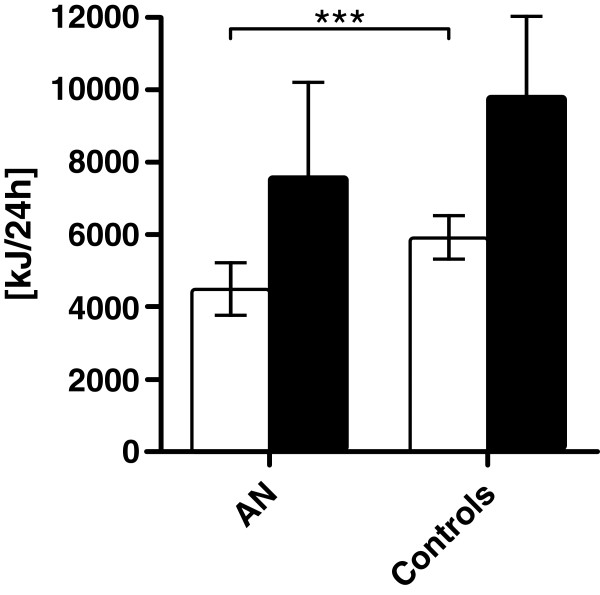
Resting energy expenditure (REE, white columns) and total daily energy expenditure (TDEE, black columns) in anorexia nervosa (n=12) and healthy controls (n=12).

**Figure 2:**
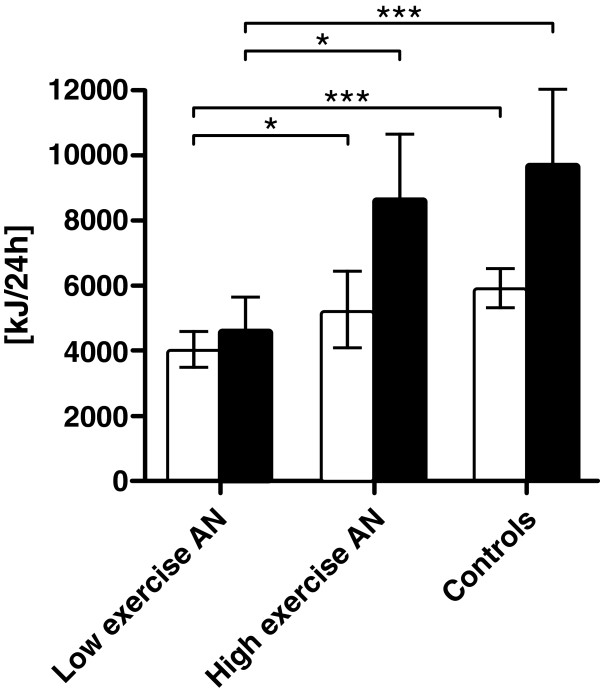
Resting energy expenditure (REE, white columns) and total daily energy expenditure (TDEE, black columns) in anorexia nervosa (AN) low-level exercisers (n=5), AN high-level exercisers (n=7), and healthy controls (n=12).

In order to gain insights into the underlying energy metabolism, REE and TDEE were assessed (Table 2). REE, determined by indirect calorimetry, was significantly decreased in patients with AN compared with healthy controls (Table 2, Figure 1). However, no significant differences in REE were found between patients and controls when adjusted for body surface area or lean body mass (LBM). Interestingly, subgroup analysis revealed that the REE of high-level AN exercisers was nearly similar to the REE of healthy controls but was significantly higher than the REE of low-level AN exercisers (Table 2, Figure 2). These relationships were also observed when the REE was adjusted for LBM or body surface area.

**Table 2:** Energy expenditure of AN compared with controls

**Variable**	**Anorexia nervosa (AN)**	**Controls**		***P***
	**n = 12**	**n = 12**		
	**M ± SD**	**M ± SD**		
Resting energy expenditure (REE, *kJ/24h*)	4457.0 ± 745.7	5885.5 ± 630.3		<0.001
REE adjusted for body surface area, SA (kJ*m^-2^*24^*-1*^)	3258.2 ± 450.9	3475.2 ± 195.2		n.s.
REE adjusted for lean body mass, LBM (kJ*kg^-1^*24h^*-1*^)	129.9 ± 16.5	135.4 ± 6.6		n.s.
Total daily energy expenditure (TDEE, *kJ/24h)*	7546.1 ± 2660.6	9741.1 ± 2237.1		0.077
	**AN High exercisers**	**AN Low exercisers**	**Controls**	
	**n = 7**	**n = 5**	**n = 12**	
	**M ± SD**	**M ± SD**	**M ± SD**	
REE *(kJ/24h)*	5218 ± 1166.9	3991.5 ± 546.8	5885,4 ± 630,3	2c, 3a,
REE adjusted for SA (kJ*m^-2^*24^*-1*^)	3761.8 ± 479.1	2968.4 ± 318.3	3475.2 ± 195.2	2c, 3b
REE adjusted for LBM (kJ*kg^-1^*24h^*-1*^)	146.0 ± 13.7	119.7 ± 12.6	135.4 ± 6.6	2b, 3b
TDEE *(kJ/24h)*	8706.9 ± 1875.7	4619,2 ± 946.7	9741.1 ± 2237.1	2c, 3a,

Values are expressed as means (M) ± standard deviations (SD). ^a^REE calculated according to the Harris-Benedict equation.

a = P<0.05, b = P<0.01, c = P<0.001, n.s. = not significant.

1 = AN high exercisers vs. controls, 2 = AN low exercisers vs. controls, 3 = AN low exercisers vs. AN high exercisers.

There was no difference in TDEE between patients with AN and controls. TDEE was also comparable in high-level AN exercisers versus controls. In contrast, the low-level AN exercisers had significantly decreased TDEE values compared to the high-level exerciser subgroup and the controls, respectively (Figure 2). The physical activity level (PAL) was 1.65, in both high-level AN exercisers and controls, whereas in low-level AN exercisers the PAL was 1.16. From the restricting subtype n=5 patients were in the low and n=3 in the high-level exerciser group. In the binge eating/purging subgroup all n=4 patients were in the high-exercise group. Next, we analyzed the plasma levels for a selected panel of hormones known to be involved in energy metabolism, including TSH, T_3_, T_4_, leptin and cortisol (Table 3). Leptin, T_3_ and T_4_ plasma levels were significantly reduced in patients with AN compared to the controls. A differential regulation was observed for T_4_, which was significantly reduced in high-level exercisers versus the controls. TSH and cortisol levels remained unchanged between the groups. For the subgroup analysis (low- versus high-level AN exercisers), no significant differences were observed. We were not able to detect a significant association between leptin levels and TDEE (partial correlation controlled for body mass index r = −0.53, p = 0.28).

**Table 3:** Comparison of hormonal levels between AN patients and controls

**Variable**	**Anorexia nervosa (AN)**	**Controls**		***P***
	**n = 12**	**n = 12**		
	**M ± SD**	**M ± SD**		
Leptin (μg/L)	1.3 ± 1.8	9.6 ± 4.7		<0.001
Cortisol (μg/L)	206.6 ± 52.9	219.6 ± 88.9		n.s.
T3 (μg/L)	0.8 ± 0.2	1.4 ± 0.4		<0.001
T4 (μg/L)	63.3 ± 15.8	80.3 ± 20.8		0.04
TSH (mU/L)	1.8 ± 0.9	1.6 ± 0.8		n.s.
	**AN ** **High exercisers**	**AN ** **Low exercisers**	**Controls**	
	**n = 7**	**n = 5**	**n = 12**	
	**M ± SD**	**M ± SD**	**M ± SD**	
Leptin (μg/L)	1.6 ± 1.9	0.9 ± 1.6	9.6 ± 4.7	1c, 2b
Cortisol (μg/L)	205.7 ± 65.2	187.1 ± 41.9	219.6 ± 88.9	
T3 (μg/L)	0.9 ± 0.3	0.8 ± 0.2	1.4 ± 0.4	1a, 2b
T4 (μg/L)	59.0 ± 9.8	71.2 ± 19.7	80.3 ± 20.8	1a

Values are expressed as means (M) ± standard deviations (SD). a = P<0.05, b = P<0.01, c = P<0.001, n.s. = not significant.1 = AN high exercisers vs. controls, 2 = AN low exercisers vs. controls, 3 = AN low exercisers vs. AN high exercisers.

Finally, we assessed psychological data of our study group by standardized and established questionnaires for depression (BDI), eating disorder (EDI-2) and eating attitude (EAT; Table 4). AN patients scored significantly higher on depression and on every subscale of the EAT and EDI-2, with the exception of the scale “maturity fears”. Additionally, the “drive for thinness” and the degree of depression were higher in the high-level AN exercisers compared with the low-level AN exercisers. The “drive for thinness” significantly correlated with TDEE (r = 0.86, p < 0.01). Moreover, in AN patients, TDEE showed a positive correlation with BDI scores (r = 0.821, p < 0.01).

**Table 4:** Psychological data of anorexic patients compared with controls

**Variable**	**Anorexia nervosa (AN)**	**Controls**		***P***
	**n = 12, M ± SD**	**n = 12, M ± SD**		
**Depression (BDI)**	21.9 ± 9.6	4.1 ± 2.9		<0.001
**Eating disorder inventory (EDI-2)**				
Body Dissatisfaction	15.2 ± 7.3	7.7 ± 8.7		<0.05
Social Insecurity	8.2 ± 4.1	1.9 ± 2.3		0.001
Interpersonal Distrust	5.1 ± 3.7	0.9 ± 1.6		0.007
Perfectionism	6.5 ± 4.2	2.0 ± 2.2		0.009
Impulse Regulation	4.5 ± 5.3	0.9 ± 1.7		*0.065*
Bulimia	2.8 ± 5.0	0.3 ± 0.6		0.027
Interoceptive Awareness	7.6 ± 4.6	0.8 ± 1.4		0.001
Maturity Fears	4.9 ± 6.0	1.7 ± 2.2		n.s.
Ineffectiveness	11.8 ± 8.0	1.2 ± 1.8		0.002
Ascetism	7.9 ± 7.2	1.9 ± 2.0		0.029
Drive for Thinness	11.7 ± 6.0	1.2 ± 2.3		<0.001
**Eating attitude test (EAT)**	52.5 ± 21.0	6.7 ± 4.1		0.001
	**AN High exercisers**	**AN Low exercisers**	**Controls**	
	**n = 7**	**n = 5**	**n = 12**	
	**M ± SD**	**M ± SD**	**M ± SD**	
**Drive for thinness (EDI-2)**	14.5 ± 3.6	9.6 ± 2.2	1.2 ± 2.3	1c, 2c, 3a
**Depression (BDI)**	24.3 ± 9.3	14.6 ± 5.6	4.1 ± 2.9	1c, 2c, 3a

Values are expressed as means (M) ± standard deviations (SD). a = P<0.05, b = P<0.01, c = P<0.001, n.s. = not significant. 1 = AN high exercisers vs. controls, 2 = AN low exercisers vs. controls, 3 = AN low exercisers vs. AN high exercisers.

## Discussion

This energy expenditure study provides new insights into the energy metabolism of patients with AN. Here we assessed, for the first time in low-level exercise and high-level exercise AN patients, compared with healthy controls 1) the differences in their energy expenditure by analyzing TDEE and REE by doubly labeled water and indirect calorimetry, respectively, 2) their hormonal status combined with 3) psychological data for depression, eating attitudes and eating disorder by established standardized questionnaires (BDI, EAT, EDI-SC, EDI-2).

We found that the daily amount of exercise in AN patients and controls was similar despite a marked difference in weight and body composition. In accordance, using the doubly labeled water method, we found no significant differences in mean TDEE between AN patients and controls. Applying the same technique, some other studies support our findings [24-26], whereas others report a reduced TDEE in AN patients [23,27,28]. We and others have found a significant reduction in REE in patients with AN compared with healthy controls [12,23,24,26,27]. This observation is mainly due to the decreased amount of LBM since we and others [23,29] found no significant differences in REE when adjusted for LBM. Besides LBM, an altered thyroid metabolism influences the REE [12,30]. In this study, TSH values were similar in AN patients and controls whereas T_3_ and T_4_ were decreased. Thus, this study supports the notion that the down regulation of the “thyrostat” in order to conserve energy is achieved by peripheral adaptive process rather than compensatory hypothalamic pituitary reaction [10,11,30]. We were not able to detect a significant association between TDEE and leptin levels. This finding is partly in contrast to other studies [31-33] and may be due to our limited sample size, and the fact that our patient sample demonstrated a heterogeneous sample of under/normal - and over exercisers. However, Holtkamp *et al.,* 2006 [32] also showed an inverted U shape association so that the authors concluded that AN hyperactivity related to hypoleptinemia cannot be conceptualized as a single mode of behavior.

Next, we performed subgroup analyses due to the marked interindividual differences in the patients regarding REE, TDEE and physical activity. Since the EDI-SC item “What percentage of your exercise is aimed at controlling your weight” was significantly correlated with TDEE, we distinguished between low-level and high-level exercisers according to this item by using a cutoff set at < 50%. There was an increased percentage of high-level exercisers in patients compared with the controls. This subgroup displayed high TDEE values and also self-reported a considerable amount of daily activity directly related at controlling their weight. The amount of daily activity in low-level AN exercisers appeared to have a decreased amount of daily activity versus the controls, this difference was not statistically significant. Similarly, Bouten *et al.*, 1996 [27] showed that AN patients were more likely to display low or high levels of activity, whereas the controls were more likely to show moderate activity levels.

Interestingly, REE in high-level exercisers was not significantly decreased versus the controls despite the decreased LBM, T_3_ and leptin plasma levels. In contrast, low-level AN exercisers displayed lower REE values compared with the high-level AN exercisers and controls, respectively, also when adjusted for LBM and body surface area. However, no differences in thyroid hormone levels were observed between the AN subgroups. In fact, two individuals of the same LBM can differ considerably in their REE due to differences in heredity and a range of physiological factors [34]. The elevated REE in high-level AN patients is possibly caused by a secondary effect resulting from the impact of physical activity on a range of physiological processes that in turn influence REE [34,35]. Additionally it is also conceivable that the body core temperatures within the AN subgroups are slightly different [9].

TDEE was comparable in high-level AN exercisers versus controls, whereas it was reduced in low-level AN exercisers. The PAL in low-level AN exercisers was only 1.16, whereas in healthy controls and in high-level AN exercisers it was 1.65. Bearing in mind that the low-level AN exercisers and controls had similar activity levels, this reflects the energy-sparing metabolic adaptations to starvation in this AN subgroup. Despite the 3-fold increased activity levels in high-level AN exercisers compared with the controls, the TDEE values were similar and thus, the energy-sparing effect, also in this AN subgroup, remarkable. Previous studies have found similar proportions of the PAL in AN patients (not distinguished between low and high activity AN patients) as we did for our high-level AN exercisers [24,27].

Hyperactivity, “drive for thinness“ and depression were increased in the AN patients as previously described [5,36]. In this study, this was particularly true for the high-level exercisers, a finding which is supported by the study of Penas-Lledo *et al.*, 2002 [37] where data on depression were retrieved from SCL-90-R scores. Consequently, this group compensated their “drive for thinness” by an increased amount of daily activity. This hyperactivity may be a component of an obsessive-compulsive behavior but could also serve to improve mood and manage stress, thus regulating negative emotions or affects [38,39]. Several studies have shown that exercise has a positive impact on a variety of psychological disorders [40,41]. Nevertheless, the increased amount of daily activity in the high-level AN exercisers was not sufficient to suppress their levels of depression to values of low-level AN exercisers or controls. In addition to the aspect and function of comorbid depression in association with pattern of high-level exercise as well as an association with binge/purge behaviour additional aspects e.g. level of impulsivity, might help to characterize AN patients to identify more homogenous endophenotypes of AN patients [42]. From an evolutionary point of view, this hyperactivity could be a result of food search behavior [43]. If in the long-term this is not rewarded by the intake of a substantial amount of food, this phenomenon could explain the increased observed depression in this subgroup.

We and others [24] did not find elevated plasma cortisol levels, although hypercortisolemia has been described in AN and in depressive patients [44]. This may be due to marked interindividual differences in cortisol levels, intraindividual stability across the day, and a range of other factors that may affect cortisol levels including physical exercise, sleep, exhaustion/fatigue, anxiety/negative effects, work hours and stress [45,46].

### Limitations of the study

The habits on activity were assessed by applying a structured interview (DDE) and not measured directly using a technique like the triaxial accelerometry. Thus, our study can not distinguish between different components of high energy expenditure e.g. high levels of exercise or micromovements. As in most studies using the doubly labeled water technique, our study population was rather small. Therefore, prospective studies with larger AN samples using both validated measures of self-reported and objectively assessed physical activity (e.g. accelerometry) combined with psychobiological aspects of starvation and reward processing might be an interesting perspective to elucidate the important aspect of hyperactivity in AN patients.

## Conclusions

Taken together, our study shows that there are marked differences in energy requirement among AN patients. Over one-half of our AN patients reported high exercise levels directly related to weight control with an overrepresentation of the binge/purge AN subgroup. Bearing in mind their dramatically increased energy expenditure and levels of depression, this subgroup may benefit from a structured treatment program specifically addressing their hyperactivity [47] supplemented where necessary with drugs derived from the group of atypical antipsychotic [48]. Studies thus far with olanzapine in AN patients have shown some promise with respect to weight gain and improvement of depression, anxiety, aggressiveness, obsessive-compulsiveness and also safety [48-50].

In order to assess the actual energy requirements and more appropriately match treatment to patients needs in AN, it is important for clinicians to quantify both energy intake and physical exercise levels. A simple and expedient way to ascertain the latter before and during treatment would be the examination of the single item “What percentage of your exercise is aimed at controlling your weight” of the EDI-SC.

## Competing interest

There is no conflict of interest in this paper for any of the authors.

## Authors’ contributions

All authors: read and approved the final manuscript. SZ: conception, design and performance of the study, acquisition of data, statistical data analysis and interpretation of data, drafted the paper. IM: interpretation of data, drafted the paper. LAB: interpretation of data. JH: laboratory analysis, interpretation of data. ST: conception and design of the study. WH: interpretation of data. SA: performance of the study. BSWD: laboratory analysis, revised the paper critically for important intellectual content. JR: conception and design of the study, revised the paper critically for important intellectual content.
